# Artificial Intelligence in Metal Additive Manufacturing: Applications in Design, Process Modeling, Monitoring, and Quality Optimization

**DOI:** 10.3390/ma19071301

**Published:** 2026-03-25

**Authors:** Juan Sustacha, Virginia Uralde, Álvaro Rodríguez-Díaz, Fernando Veiga

**Affiliations:** 1 Department of Engineering, Public University of Navarre, Campus of Arrosadía, 31006 Pamplona, Spain; sustacha.174559@e.unavarra.es (J.S.); or ard@edu.xunta.gal (Á.R.-D.); 2 Department of Engineering, Public University of Navarre, Campus of Tudela, 31500 Tudela, Spain; virginia.uralde@unavarra.es; 3 Department of Transportation and Maintenance of Vehicles, CIFP Fontecarmoa, 36600 Vilagarcía de Arousa, Spain

**Keywords:** metal additive manufacturing, artificial intelligence, machine learning, deep learning, DfAM, process monitoring, defect detection, digital twin, physics-informed modeling

## Abstract

**Highlights:**

**What are the main findings?**

- AI supports DfAM, parameter selection, monitoring, and certification in metal AM.
- ML/DL can predict defects, distortion, and properties from in situ and ex-situ data.
- Hybrid physics–ML models and digital twins are the most promising scalable approach.

**What are the implications of the main findings?**

- Faster parameter qualification can reduce trial-and-error, scrap, and post-processing.
- Closed-loop control with multi-sensor fusion can improve consistency and reliability.
- Standard datasets, uncertainty quantification, and validation are key for adoption.

**Abstract:**

Metal additive manufacturing (MAM) enables the production of complex, high-value components for sectors such as aerospace, energy, and biomedical engineering. However, its large-scale industrial adoption remains constrained by internal defects, residual stresses, distortions, microstructural variability, and the complexity of the coupled process-parameter space. This review examines how artificial intelligence (AI)—including machine learning, deep learning, and optimization algorithms—is being applied to address these challenges across the MAM workflow. A structured literature review was conducted covering studies published between 2015 and 2025, identified through searches in Scopus, Web of Science, and IEEE Xplore. The selected literature is analyzed according to key functional domains of metal additive manufacturing: design for additive manufacturing (DfAM), process modeling and simulation, in situ monitoring and control, and microstructure and property prediction. AI approaches are further categorized by learning paradigm, including supervised learning, deep learning, reinforcement learning, and hybrid physics–machine learning models. The review highlights recent advances in AI-assisted parameter optimization, defect detection, and digital-twin frameworks for process supervision. At the same time, it identifies persistent challenges, particularly the scarcity and heterogeneity of datasets, limited transferability across machines and materials, and the need for uncertainty-aware models capable of supporting validation and certification. Overall, the analysis indicates that the integration of multi-sensor monitoring with hybrid physics-informed AI models represents the most promising near-term pathway to improve process reliability, reduce trial-and-error experimentation, and accelerate industrial qualification in metal additive manufacturing.

## 1. Introduction

In recent years, Metal Additive Manufacturing (MAM) has moved beyond the status of an emerging technology to become a strategic resource in high–value-added industrial sectors such as aerospace [1], automotive [2], energy [3], and biomedicine [4]. Its ability to fabricate complex, customized geometries with optimized material usage distinguishes it from many conventional manufacturing processes (e.g., machining, casting, forging, stamping, or injection-based forming), where efficiency and design flexibility are often constrained by material removal requirements, tooling limitations, or process-specific geometric restrictions [5,6].

Despite its considerable potential, MAM faces significant technical challenges [7,8]. Most relevant challenges include the occurrence of internal defects (porosity, cracks), the generation of residual stresses and distortions due to thermal gradients, the complexity of predicting microstructures, and the need to optimize process parameters (speed, power, scanning strategy, material feed, etc.) [9]. These interdependent variables define an extremely large design space that is difficult to explore using traditional approaches.

In this context, Artificial Intelligence (AI) emerges as a key tool to overcome these limitations [10]. Through Machine Learning (ML) [11] and Deep Learning (DL) [12], it becomes possible to analyze large volumes of experimental and monitoring data, develop predictive models that are faster and more accurate than traditional physics-based methods, and apply real-time adaptive control strategies to ensure process quality. In addition, AI enables the integration of simulation, topology optimization, and generative design, thus promoting a more intelligent and efficient Design for Additive Manufacturing (DfAM) approach [13].

This article presents a state-of-the-art review of the application of AI to MAM, analyzing the main current research lines, the results achieved, and the remaining challenges. It also proposes a classification of AI applications according to their impact on design, simulation, process control, and material characterization, in order to provide an integrated view of the transformative potential of artificial intelligence in the evolution of metal additive manufacturing.

Unlike previous reviews that tend to focus on specific techniques (e.g., monitoring or topological optimisation) or general AI trends in additive manufacturing, this work provides a structured, application-oriented synthesis of artificial intelligence in the main stages of the metal additive manufacturing workflow. The contribution of this review lies in organising current research according to functional domains (design, simulation, monitoring/control, and property characterisation) and learning paradigms (supervised learning, deep learning, reinforcement learning, and hybrid physics–ML models), enabling a consistent cross-comparison of approaches. Particular emphasis is placed on the role of hybrid modelling and multisensor integration as mechanisms for bridging the gap between research prototypes and industrial deployment. By explicitly discussing data-related limitations, transferability challenges, and certification constraints, this review aims to provide a consolidated and practical perspective for researchers and practitioners seeking to integrate AI into metal additive manufacturing.

This review follows a structured literature review protocol designed to improve transparency and reproducibility. The bibliographic search was conducted in the Scopus, Web of Science, and IEEE Xplore databases, covering the period from 2015 to 2025. The search strategy combined terms related to metal additive manufacturing and artificial intelligence, including “metal additive manufacturing”, “machine learning”, “deep learning”, “digital twin”, “process monitoring”, and “defect prediction”, using different Boolean combinations depending on the database.

After retrieval, duplicate records were removed and the remaining studies were screened in two stages. First, titles and abstracts were evaluated to determine their relevance to the scope of this review. Second, the full texts of the preselected papers were examined for eligibility. Studies were included when they addressed the application of artificial intelligence, machine learning, deep learning, reinforcement learning, or hybrid physics-informed data-driven approaches to metal additive manufacturing, with relevance to at least one of the following domains: design for additive manufacturing, process modeling and simulation, in situ monitoring and control, or microstructure and property prediction. Studies focused exclusively on polymer additive manufacturing, lacking methodological transferability to metallic processes, were excluded. Reviews, editorials, and papers with insufficient technical detail were also excluded from the final synthesis.

**Figure 1:**
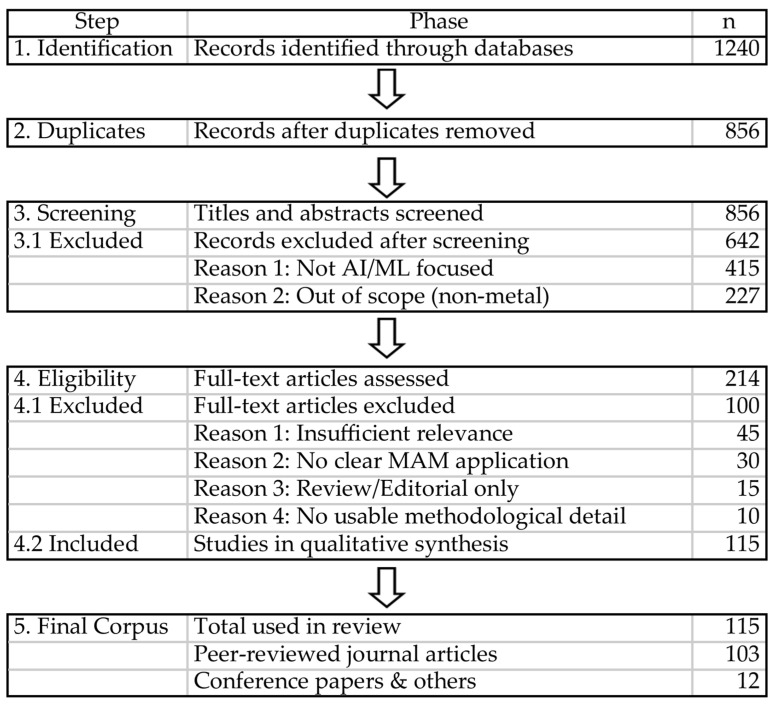
PRISMA-style flow diagram of the literature selection protocol used in this review.

To improve methodological transparency and reduce selection bias, this review adopted a structured literature review approach. Structured review methodologies are widely recommended when the objective is to identify, screen, and synthesize a broad body of literature in a reproducible and traceable manner, particularly in interdisciplinary fields where terminology, application domains, and methodological traditions may vary substantially [14]. In this context, the use of an explicit search strategy, predefined eligibility criteria, and a flow-based reporting structure helps strengthen the rigor of the review process and improves the clarity with which the final corpus is justified. Accordingly, the present study was designed following the principles of systematic review reporting and documented through a PRISMA-style selection flow [15], adapted to the scope of an engineering-focused review. Following this procedure, 115 articles were retained for full-text assessment, resulting in a final corpus composed of 103 journal articles and 12 conference papers. Figure 1 summarizes the selection workflow in PRISMA-style form.

While several previous review papers have addressed artificial intelligence in additive manufacturing, many of them either examine additive manufacturing in a broad cross-material sense or focus on specific subtopics such as defect detection, monitoring, topology optimization, or data-driven process prediction. As a result, the literature still lacks a review that integrates the main artificial intelligence approaches specifically within the workflow of metal additive manufacturing and discusses them not only from an algorithmic perspective, but also from the standpoint of industrial maturity and qualification requirements.

In this context, the differentiating contribution of the present review is threefold. First, it is specifically centered on metal additive manufacturing, whose process physics, defect mechanisms, monitoring requirements, and certification constraints differ substantially from those of polymer-based systems. Second, it organizes the literature through a dual perspective: by functional domain within the MAM workflow (DfAM, process modeling and simulation, monitoring/control, and microstructure/property prediction) and by learning paradigm (supervised learning, deep learning, reinforcement learning, and hybrid physics–ML approaches). Third, beyond summarizing published applications, the review provides a maturity-aware and deployment-oriented synthesis, explicitly discussing transferability, multisensor integration, validation against ex situ evidence, uncertainty, and certification-related limitations. In this sense, the goal of the paper is not only to compile existing studies, but to clarify the current position of AI in MAM and identify the conditions required for its robust industrial adoption.

Accordingly, the objective of this review is not only to summarize recent applications of artificial intelligence in metal additive manufacturing, but also to analyze them across the main functional stages of the MAM workflow and to assess their current level of maturity, validation, transferability, and relevance for industrial deployment. From this perspective, the review is structured to connect algorithmic developments with practical manufacturing requirements, so that the discussion and conclusions reflect not only what has been achieved in the literature, but also what remains necessary for robust and certifiable implementation. The analysis focuses on four key domains of the MAM workflow: design for additive manufacturing (DfAM), process modeling and simulation, monitoring and control, and microstructure and property prediction. In addition, the reviewed approaches are categorized according to their learning paradigm, including supervised learning, deep learning, reinforcement learning, and hybrid physics–machine learning models. This structure is intended to clarify current research trends, identify technological limitations, and highlight future directions for the integration of AI in metal additive manufacturing.

## 2. Fundamentals of Metal Additive Manufacturing

**Figure 2:**
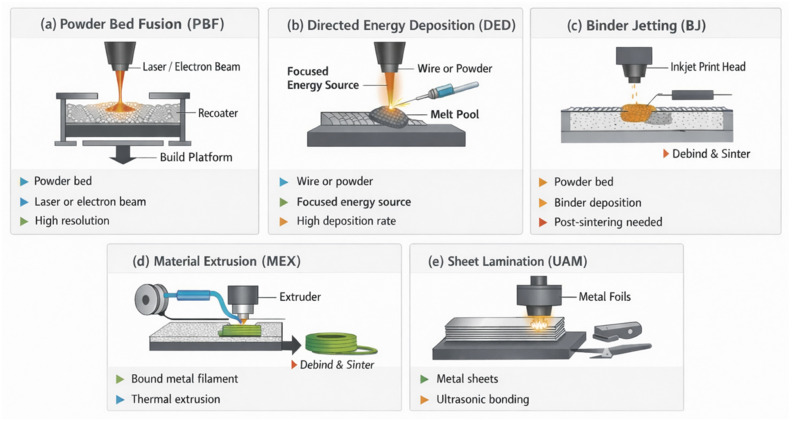
Schematic overview of the main metal additive manufacturing process families considered in this review: (**a**) Powder Bed Fusion (PBF), (**b**) Directed Energy Deposition (DED), (**c**) Binder Jetting (BJ), (**d**) Material Extrusion (MEX), and (**e**) Sheet Lamination/Ultrasonic Additive Manufacturing (UAM).

Unlike conventional methods based on subtractive manufacturing or forming, MAM provides significant advantages in terms of design freedom, waste reduction, and customization capabilities. However, large-scale implementation still faces important technical and economic challenges that require innovative solutions. To contextualize these challenges, it is useful to distinguish the main metal additive manufacturing (MAM) process families, as it can be seen in Figure 2, since each offers a different balance between resolution, productivity, build size, and cost [16]. Within MAM, several key technologies address different production needs:

- Powder Bed Fusion (PBF-LB/M or PBF-EB/M): This group includes laser-based powder bed fusion (Laser Beam Powder Bed Fusion, PBF-LB/M) [17] and electron-beam powder bed fusion (Electron Beam Powder Bed Fusion, PBF-EB/M) [18]. Both rely on depositing thin layers of metal powder that are selectively melted by an energy source. These technologies stand out for high geometric resolution and excellent surface finish, making them suitable for producing parts with high dimensional accuracy and superior mechanical properties. Their main limitations are reduced build volume, high costs associated with laser or vacuum systems, and limited productivity compared to other methods [19].
- Directed Energy Deposition (DED): This family includes technologies such as Wire Arc Additive Manufacturing (WAAM or DED-ARC/W) [20] and Laser-Metal Deposition (LMD or DED-LB/M) [21] with metal powder. Unlike powder bed processes, here the material (wire or powder) is delivered in a focused manner onto the part and melted by an energy source (laser, electron beam, or electric arc). The main attraction of DED—and particularly DED-ARC/W—is its high deposition rate, enabling faster production of large components at relatively low cost. However, these technologies offer lower geometric resolution and often require post-machining to reach adequate tolerances and finishes [22].
- Binder Jetting and hybrid technologies: Although less mature in metals, Binder Jetting and hybrid processes combined with CNC machining are gaining interest. They enable rapid production of complex geometries with higher productivity than powder bed technologies, although mechanical properties may be lower due to the need for additional sintering or infiltration steps [23].
- Material Extrusion (MEX/Bound Metal Deposition): Metal material extrusion technologies deposit a filament or pellet feedstock composed of metal powder bound in a polymer matrix, which is subsequently subjected to debinding and sintering to obtain a dense metallic component. These processes are gaining attention due to their lower equipment cost and accessibility compared with powder bed fusion systems. However, dimensional shrinkage during sintering and the control of densification remain important challenges that affect final accuracy and mechanical properties [24,25].
- Sheet Lamination (Ultrasonic Additive Manufacturing–UAM): Sheet lamination processes build components by bonding thin metal sheets layer by layer, typically through ultrasonic welding, followed by machining to achieve the final geometry. This approach enables the fabrication of multi-material structures and the integration of embedded sensors or cooling channels during manufacturing. Nevertheless, the achievable geometric complexity and mechanical bonding quality between layers remain areas of ongoing research [26,27,28].

Table 1 summarizes the main metal additive manufacturing technologies and their typical industrial applications. It should be noted that these applications are not exclusive to each process; depending on material compatibility, machine configuration, and process parameters, several technologies can be applied across multiple sectors such as aerospace, biomedical, or energy. Powder bed fusion processes (PBF-LB/M and PBF-EB/M) provide the highest dimensional accuracy and density, which explains their prevalence in high-value applications such as aerospace and biomedical components, albeit with limitations in build volume and higher equipment/operational costs. In contrast, directed energy deposition routes (DED) and particularly wire-based DED-ARC/W prioritize deposition rate and scalability for large parts, at the expense of geometric resolution and with a stronger reliance on post-processing. Finally, Binder Jetting stands out for productivity and support-free fabrication of complex geometries, but typically requires sintering/infiltration steps that may penalize final mechanical performance, positioning it for medium-series production and tooling applications.

### 2.1. Advantages and Technical Challenges of MAM

From a process engineering perspective, metal additive manufacturing presents specific advantages and limitations that differ significantly across the main technology families. Among its main benefits are design freedom, which enables the production of highly complex geometries that are not feasible by machining or traditional casting; optimized material usage, resulting in reduced waste and lower costs associated with machining; and the possibility of customization and on-demand production, which is strategic in sectors such as biomedical, aerospace, and energy. In addition, technologies such as DED-ARC/W or DED-LB/M facilitate the repair and coating of components, extending the service life of critical parts and providing clear added value.

However, MAM also faces a series of technical challenges that limit large-scale adoption. These include the management of internal defects (porosity, cracks, lack of fusion), the generation of residual stresses and thermal distortions—especially in high-temperature layer-by-layer deposition processes—and variability in mechanical properties, which complicates certification in regulated sectors. Moreover, reproducibility and scalability remain challenging, as final quality depends on multiple process parameters with nonlinear interactions, increasing control complexity. In parallel, the cost of equipment, metal powders, and post-processing remains high, reducing competitiveness compared to other manufacturing technologies.

In this context, the need for advanced monitoring, modeling, and optimization tools becomes evident, where AI is positioned as a strategic ally. Its integration can enable early defect detection through computer vision, prediction of thermal distortions with machine-learning models, and automatic optimization of process parameters—helping to overcome current obstacles and consolidate MAM as a mature and reliable technology. Table 2 summarizes the main advantages and technical challenges associated with metal additive manufacturing processes. It should be noted that some phenomena, such as oxidation risk, depend strongly on the material being processed and the process atmosphere rather than being inherent to a specific additive manufacturing technology. From a design standpoint, MAM offers unprecedented geometric freedom and enables complex, topology-optimized structures [38]. Regarding performance, high density and strength are routinely achievable; however, microstructural variability and internal defects (e.g., porosity and cracking) remain critical concerns. At the process level, on-demand production and repair/coating capabilities are attractive, but robust control is challenged by strong parameter coupling, thermal gradients, residual stresses, and distortion. In addition, several process variables in metal additive manufacturing are strongly influenced by the material being processed. Factors such as heating kinetics, atmosphere requirements, homogeneity of deposition temperature, and cooling rates depend on the thermophysical and metallurgical characteristics of the alloy system. Consequently, predictive models and AI-based optimization strategies must account for these material-dependent effects to ensure reliable process control and accurate property prediction [34,39,40]. Finally, although MAM is already impactful in biomedical and other safety-critical sectors, the absence of consolidated standards and certification pathways, together with high equipment and post-processing costs, continues to be a major barrier to scaling beyond high-value niches. Finally, although MAM is already impactful in biomedical and other safety-critical sectors, the absence of consolidated standards and certification pathways, together with high equipment and post-processing costs, continues to be a major barrier to scaling beyond high-value niches.

In addition to PBF-LB/M, PBF-EB/M, DED, and WAAM, it is also worth considering indirect metal additive manufacturing routes based on feedstock deposition followed by debinding and sintering, including bound-metal or catalytic MEX-type technologies. Although less represented in the AI literature, these processes are relevant because of their lower equipment complexity and potential accessibility. Their main challenges differ from fully dense fusion-based routes and include shrinkage prediction, densification control, defect evolution during debinding/sintering, and final-property variability, which makes them suitable candidates for AI-assisted prediction and compensation strategies [41]. In some deposition-based metal additive manufacturing processes, the interaction between the deposited material and the build substrate can also influence defect formation. In particular, the use of ceramic interlayers or release coatings (e.g., boron nitride or other ceramic separation layers) to facilitate part removal from the build plate may modify the thermal boundary conditions during deposition. This can affect heat dissipation, residual stress development, and consequently the occurrence of distortion or cracking in the fabricated component [34,42].

**Table 2:** Advantages and technical challenges of Metal Additive Manufacturing.

Aspect	Advantages	Technical Challenges
Design	Full geometric freedom, complex structures, topology optimization [43].	Limitations in dimensional accuracy and surface finish for certain technologies (e.g., DED-ARC/W, DED-LB/M) [44,45].
Materials	Reduced material waste vs. machining. Use of recyclable metal powders and wires.	High cost of raw materials. Possible oxidation or contamination depending on alloy sensitivity and processing atmosphere [1,46].
Mechanical properties	High density and strength in PBF-LB/M and PBF-EB/M. Lightweight and functional structures [39].	Microstructural variability. Internal defects (porosity, cracks) [47].
Processes	On-demand production. Repair and coating of existing parts [48].	Complexity of parameter control. Thermal distortions and residual stresses [49].
Applications	Custom biomedical components. Critical parts in aerospace, energy, and automotive [50].	Lack of consolidated standards and certification frameworks for industry [51].
Economics	Reduced lead times and inventory.	High costs of equipment, post-processing, and maintenance [52].

### 2.2. Potential of Artificial Intelligence in MAM

**Figure 3:**
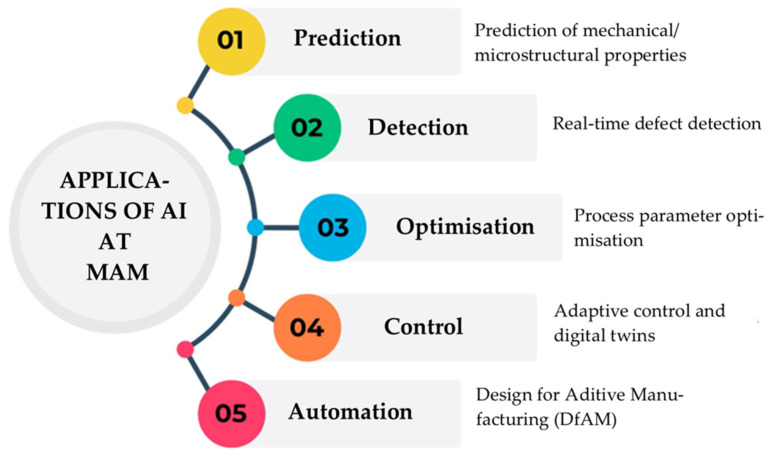
Applications of artificial intelligence at metal additive manufacturing.

The complexity of the physical phenomena involved in Metal Additive Manufacturing—such as rapid solidification, heat transfer, residual stress generation, or the formation of internal defects—together with the large number of interdependent process parameters (laser power, scanning speed, material flow rate, deposition path, among others), has generated growing interest in applying AI as a support and optimization tool. ML and DL algorithms offer solutions ranging from process-parameter optimization—reducing the time required by traditional trial-and-error methods—to early real-time defect detection through computer vision systems and thermal or acoustic sensors, reinforcing quality control [40]. AI also enables prediction of mechanical and microstructural properties from experimental data, complementing or even replacing physics-based simulations that are often costly in time and computational resources, as it can be seen on Figure 3. Another fundamental contribution lies in the development of digital twins, capable of representing the real process behavior and providing adaptive control strategies, increasing the reliability and robustness of production. Finally, AI-assisted DfAM automation through topology optimization and generative design opens new perspectives for highly efficient and customized components. Consequently, integrating AI and MAM not only increases process efficiency and consistency, but also lays the foundations for smart manufacturing within Fourth Industrial Revolution, where systems learn, optimize, and adapt autonomously.

## 3. Overview of Artificial Intelligence in Engineering and Manufacturing

In this review, AI is considered from a functional rather than purely taxonomic perspective. The methods most relevant to metal additive manufacturing are supervised learning models for process–property prediction, deep-learning architectures for image and signal analysis, reinforcement learning for adaptive decision-making, and hybrid physics–ML approaches for reduced-order modeling and digital twins. These families are discussed throughout the manuscript in relation to their role in design, simulation, monitoring, and qualification rather than as isolated algorithmic categories.

Within this framework, several AI approaches are particularly relevant for engineering applications:

- Machine Learning (ML): algorithms capable of identifying patterns and hidden relationships in large volumes of data, enabling prediction and classification without explicitly programming every system rule.
- Deep Learning (DL): a subfield of ML based on deep neural networks, especially useful for processing images, signals, and unstructured data, making it a key tool for real-time monitoring of industrial processes.
- Artificial Neural Networks (ANN): structures inspired by biological nervous systems, capable of approximating complex nonlinear functions and modeling complex physical phenomena.
- Evolutionary and metaheuristic algorithms: techniques inspired by natural processes such as genetic selection or evolution, enabling the solution of multidimensional optimization problems, such as process-parameter allocation or complex geometry design.

In manufacturing environments, these approaches are commonly used to analyze experimental and sensor data, develop predictive models, and support adaptive process optimization.

## 4. AI Applications in Metal Additive Manufacturing

Artificial intelligence is transforming industry; in particular, in additive manufacturing it is addressing inherent process challenges and unlocking potential across the entire product life cycle. This technological synergy enables design optimization, prediction of material behavior, and quality assurance in ways that traditional methods cannot match.

### 4.1. Design Optimization for MAM (DfAM)

At the design stage, AI is transforming DfAM, breaking with the limitations of traditional methodologies and enabling new ways to explore the geometric design space. Several recent studies and reviews show how AI methods are being integrated across multiple stages of additive manufacturing (AM), from conceptual geometry generation to in-process control, driven by the complexity of the design space and the inherent variability of manufacturing outcomes [53,54]. These works also highlight, alongside opportunities, the presence of terminological inconsistencies in the field, reinforcing the need for standardization in evaluation and conceptual frameworks [55,56].

Generative design is a paradigmatic example of this transformation. It is not merely a support tool, but an approach in which AI algorithms autonomously create complex geometries from requirements and manufacturing constraints. The resulting solutions often take organic, nature-inspired forms that are lighter and more efficient than conventional parts. A representative case is the redesign of structural brackets in aerospace, where AI has enabled weight reductions of up to 50% without compromising strength, directly reducing fuel consumption and improving operational efficiency.

Complementarily, AI-assisted topology optimization takes this concept further. Algorithms analyze an existing part and intelligently remove material that does not contribute to structural strength, preserving only the essential geometry. When combined with metal additive manufacturing, the goal is not only weight reduction, but also support minimization, reduced internal stresses, or improved heat dissipation—key aspects for critical components such as heat exchangers or customized medical implants.

From a methodological perspective, several classes of algorithms have been identified as especially effective for specific tasks. Supervised models and ensembles (such as artificial neural networks, support vector machines, random forests, or XGBoost) have shown high predictive accuracy for mechanical properties and surface quality [56]. Deep-learning models (CNN, GAN) are widely used for shape generation and topology synthesis under constraints, although they often require additional filtering mechanisms to ensure manufacturing feasibility [57,58]. Reinforcement learning is emerging for high-dimensional sequential problems such as deposition path planning, providing innovative solutions when dense reward functions are designed [59]. In addition, surrogate modeling and bidirectional networks enable mappings between design and properties, allowing inversion of the process to generate geometries meeting target performance while reducing the cost of extensive simulations [55].

The literature also reports hybrid applications combining AI-based predictors with evolutionary or metaheuristic algorithms to address multi-objective optimization problems in DfAM [60]. These approaches have shown advantages over traditional methods such as Taguchi designs of experiments, achieving better accuracy in property prediction or process-parameter selection [61]. Significant advances have also been documented in quality prediction and real-time defect identification, using CNNs for images or supervised ensembles for in situ monitoring in metallic and polymer AM processes [62].

Nonetheless, challenges remain that limit broad adoption. Key issues include scarce and heterogeneous open datasets, limited fidelity of generated geometries when manufacturability constraints are not explicitly enforced, and the need for hybrid models integrating physics and machine learning to improve extrapolation capability [57,63]. Interpretability is also a critical barrier in regulated domains, motivating the inclusion of explainability techniques and uncertainty quantification [56]. Table 3 summarizes the main AI applications in AM, linking each problem class to representative algorithms and selected results reported in the literature.

AI-driven DfAM remains more mature as a support tool for exploration and optimization than as a fully autonomous design route. Generative and topology-oriented models can accelerate design-space exploration, but their industrial usefulness still depends on whether manufacturing feasibility constraints, process variability, and post-processing requirements are explicitly incorporated. Therefore, the main limitation is not geometric generation itself, but the gap between digitally optimal solutions and solutions that remain robust once produced, inspected, and qualified in metal AM environments.

### 4.2. Simulation and Process Modeling

Process simulation in MAM is crucial to predict and mitigate defects, thermal distortions, and residual stress development arising from the highly coupled thermo-mechanical and metallurgical phenomena inherent to layer-by-layer metal deposition [65]. In this context, AI models offer a faster and more flexible alternative to traditional physics-based simulations [66,67]. These predictive models, often based on machine learning, are trained with manufacturing data to anticipate critical variables—such as residual deformation, temperature gradients, or microstructural evolution—with high accuracy [68,69]. While physical simulations such as the Finite Element Method (FEM/FEA) provide deep insight into underlying phenomena and high-fidelity predictions, their high computational cost can extend runtimes to hours or days on supercomputers [70,71,72].

In contrast, AI models can deliver inference times from milliseconds to seconds after training, making them particularly suitable for online monitoring and real-time control [73,74,75]. Although these models lack implicit physical constraints, their generalization capability depends strongly on the representativeness and quality of the training data [76,77]. An emerging solution is the hybrid approach, where AI is used for rapid initial prediction and physics-based simulations are used for final validation, achieving a balance between accuracy and speed [78,79].

Recent advances in AI-based defect prediction demonstrate strong potential for real-time quality monitoring. The use of convolutional neural networks (CNN) applied to layer-by-layer images enables detection of delaminations and interlaminar imperfections with accuracies above 95%, reducing the need for human intervention [66,80]. Similarly, transfer learning with pretrained architectures such as AlexNet has shown outstanding performance in multi-defect classification in FDM parts after hyperparameter optimization [76]. Edge deployment (e.g., NVIDIA Jetson Nano) enables online inspection of beads and joints, supporting early parameter adjustment during printing [67]. Finally, multi-sensor fusion approaches—combining thermal, vibration, and image data—significantly outperform unimodal models, yielding more robust and reliable classifications [81].

The debate regarding advantages and limitations of physics-based simulations versus AI models remains open. On the one hand, FEM/FEA models remain essential for detailed analysis of thermal gradients and residual stresses, provided mesh quality and experimental validation are adequate [70,71]. However, the complexity of transient thermal simulations or melt-flow models limits their use to offline analysis due to prohibitive computational costs for real-time applications [71,72]. On the other hand, AI-based models can be deployed for online control, though their accuracy depends on the data domain on which they were trained [73,76,77].

Hybrid approximations and reduced-order models (ROM) represent a promising middle path. Physics-informed models combined with machine learning can reproduce physics-model outputs at a fraction of the computational cost, especially when deployed on GPU-accelerated architectures [82,83,84]. Examples include AI-based phase-field models capable of reproducing microstructural evolution with high accuracy relative to reference simulations, but with substantial runtime savings [85]. Likewise, digital twin frameworks integrating multi-sensor data with machine learning enable near-real-time monitoring and corrective actions during the process [78,86].

The most relevant advances in predicting critical variables illustrate this paradigm shift. ML models specialized in geometric distortion can predict residual deformation and warping, enabling proactive adjustments before defects manifest [66,87]. Similarly, AI-based thermal models trained on physics simulations can rapidly estimate temperature fields, supporting real-time thermal management strategies [88]. Finally, physics-informed neural networks show strong potential to accelerate 3D microstructural evolution simulations without sacrificing accuracy relative to reference models [85]. Table 4 highlights key differences between traditional physics-based methods and AI models in terms of accuracy, computational resources, and applicability to online control. In this manuscript, hybrid models are considered as approaches that combine physics-based simulations with data-driven methods. These include physics-guided models, where physical constraints or governing equations are embedded in machine-learning architectures; ML-accelerated models, where machine learning acts as a surrogate for computationally expensive simulations; and ML-augmented models, which combine experimental or sensor data with simplified physical models to improve prediction of process–structure–property relationships.

Mechanical-property prediction in additive manufacturing should also be framed relative to analytical and mechanistic baselines. In lattice or architected structures, models such as Gibson–Ashby remain useful for first-order stiffness scaling, while defect-based approaches such as Tanaka–Murakami can provide physically interpretable links between defect populations and strength reduction. However, in practical AM components, final properties are influenced simultaneously by porosity, microstructure, residual stress, surface state, build orientation, thermal history, and post-processing, which limits the predictive reach of simplified analytical models when used alone. In this context, recent literature suggests that nonlinear machine-learning models and neuro-fuzzy approaches may capture these interactions more effectively, although their outputs still benefit from being interpreted against physically meaningful baselines rather than as purely black-box estimates. Prada Parra et al. reported [89], in the context of additively manufactured composites, that supervised learning approaches improved the prediction of complex mechanical responses relative to simpler alternatives, whereas Sagias et al. [90], showed that ANFIS-based models can be effective for mechanical-property prediction under nonlinear, multi-parameter AM conditions. Although these studies are not direct evidence for metal AM in all cases, they reinforce the broader methodological argument that data-driven models become especially valuable when the property response depends on coupled variables that are difficult to isolate analytically.

**Table 4:** Comparison between physics-based simulations and AI models in MAM.

Aspect	Physics-Based Simulations (FEM/FEA)	AI/Machine Learning Models	Hybrid Approaches
Accuracy	Very high with good meshing and experimental validation [70,71]	Dependent on training data quality and representativeness [76,77]	High, combining fast prediction with physical validation [78,79]
Computational cost	High: hours or days on HPC for full simulations [72]	Low after training: predictions in seconds or milliseconds [73,75]	Medium: orders-of-magnitude reduction via ROM and GPUs [83,84]
Real-time applicability	Limited to offline analysis; not viable for online control	High: suitable for online monitoring and parameter tuning [67,73]	Very high: suited for closed-loop control and digital twins [78,86]
Limitations	Sensitive to mesh quality; high simulation cost [71]	Overfitting risk; poor extrapolation if data are not representative [91,92]	Requires hybrid datasets and continuous experimental validation [93]

These trends point to a future where AI does not merely complement, but amplifies traditional simulation capabilities. Integrating fast predictive models with selective physical validation enables autonomous manufacturing environments, where process parameters can be continuously optimized from real-time feedback [67,78]. However, important challenges remain, including the need for representative and standardized databases across materials, geometries, and printer configurations [91,92], and the integration of validation loops based on post-process metrology (e.g., CT and mechanical testing) to ensure robustness and reliability [93].

From a critical perspective, the most credible near-term role of AI in simulation and process modeling is not to replace physics-based approaches, but to extend their practical usability across broader parameter spaces and shorter decision times. In metal additive manufacturing, where process responses are highly sensitive to material condition, geometry, machine configuration, and thermal history, purely data-driven models remain vulnerable to limited transferability and domain shift. Consequently, the greatest value is likely to come from hybrid strategies in which AI accelerates prediction, filtering, or optimisation, while physics-based models and experimental validation preserve interpretability and reliability. Under this view, the future of process modeling in MAM lies not in choosing between FEM and AI, but in combining both within validated, uncertainty-aware workflows capable of supporting industrial decision-making.

It should be noted that artificial intelligence does not replace the fundamental physical understanding of metal additive manufacturing processes. Phenomena such as phase transformations, solidification behavior, crystallographic structure, alloy chemistry, and hardenability play a fundamental role in determining the final microstructure and mechanical properties of additively manufactured components. In addition, geometric factors such as part size, volume, and thermal mass influence heat transfer conditions and solidification dynamics during fabrication. Therefore, AI-based models are most effective when combined with physical metallurgy knowledge and process simulations, enabling hybrid approaches that integrate data-driven prediction with physically grounded understanding [94,95].

### 4.3. Quality Control and Real-Time Monitoring

Quality control is one of the main bottlenecks in metal additive manufacturing, and this is precisely where AI has shown some of its most tangible short-term benefits. In practice, monitoring systems may rely on layerwise images, melt-pool videos, thermographic maps, photodiode signals, acoustic emissions, or combinations of these streams [96]. Convolutional neural networks (CNNs) are especially relevant when the input is spatially structured, such as powder-bed images or thermal fields, because they can automatically extract defect-related features without manual feature engineering. For example, thermographic monitoring in laser powder bed fusion has been used to train deep-learning models for in situ defect detection [97], while image-based regression CNN approaches have also been explored for porosity estimation from inspection-linked datasets [98]. Likewise, photodiode-based monitoring has been investigated as a lightweight route for process tailoring and quality prediction in LPBF, especially when real-time deployment constraints make full-frame imaging impractical [99].

From a practical standpoint, the main distinction is not only between algorithms, but between sensing strategies. Vision and thermography provide rich spatial information and are therefore effective for detecting spatter, lack-of-fusion signatures, melt-pool instability, or anomalous thermal patterns. However, they generate large data volumes and may require careful calibration, line-of-sight control, and substantial annotation effort. In contrast, photodiodes and acoustic signals are easier to integrate and cheaper to process, but they are usually more indirect and require stronger modeling assumptions to link signals with part-quality outcomes. For this reason, recent work increasingly favors multi-sensor fusion, which combines complementary modalities to improve robustness and reduce false positives. Studies combining heterogeneous sensing streams with deep learning, as well as optical and acoustic “qualify-as-you-go” approaches, illustrate this trend toward more reliable monitoring architectures [100].

**Figure 4:**
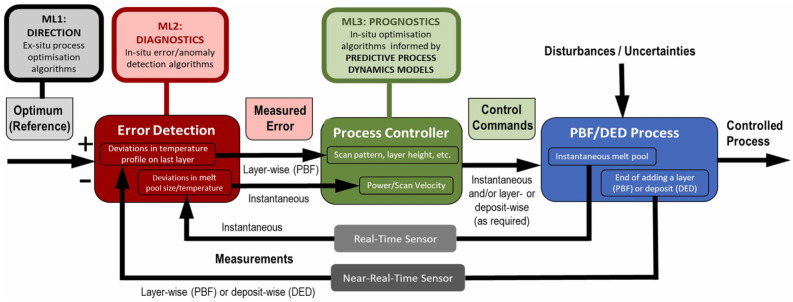
Representative AI-enabled architecture for in situ monitoring and closed-loop process control in metal additive manufacturing. (Extracted from [101] licensed under CC BY 4.0.).

Representative architecture for AI-enabled in situ monitoring and closed-loop process supervision in metal additive manufacturing [101]. The scheme, Figure 4 illustrates three complementary levels of machine-learning integration: ML1 for ex situ process optimisation and reference generation, ML2 for in situ error and anomaly detection based on real-time or near-real-time measurements, and ML3 for prognostics and predictive control. Sensor-derived information is used to identify deviations in melt-pool behavior, temperature, or layer/deposit evolution, which are then transferred to the process controller to adjust parameters such as scan pattern, layer height, power, or scan velocity. The framework also highlights the influence of disturbances and uncertainties and the distinction between instantaneous and layer-/deposit-wise decision loops in PBF and DED processes.

Finding the process-parameter set (laser power, scanning speed, deposition strategy) required to obtain a high-quality part is a formidable challenge due to interaction complexity. AI provides powerful solutions for multi-objective optimization. Genetic algorithms can be used to automatically explore many parameter combinations, “evolving” the best sets based on fitness objectives such as density, hardness, or surface roughness [102,103]. In addition, reinforcement learning allows the system to learn how to make parameter decisions in real time. Acting as an agent, AI receives a “state” (sensor data) and takes an “action” (adjusting a parameter) to maximize a “reward” (part quality). For example, the agent could learn to intelligently modulate laser power to avoid thermal stresses [104]. Despite this rapid progress, the industrial deployment of AI-driven inspection in metal additive manufacturing remains uneven. MAM offers a clear advantage in data availability—modern systems provide rich, synchronized streams (melt-pool monitoring, thermal imaging, acoustic emissions, photodiodes, layerwise vision, machine logs)—yet these signals do not automatically translate into robust, transferable models. In practice, most published approaches are demonstrated on limited datasets, controlled conditions, and a narrow set of geometries/materials, with scarce evidence of stable performance under production variability (powder lots, machine-to-machine drift, optics contamination, recoater wear, build layout effects) or across multiple sites. Moreover, inspection is still frequently treated as “defect detection” rather than end-to-end quality assurance: the traceability between in-process indicators and certified part acceptance (e.g., CT/UT outcomes, mechanical allowables, and standards-based qualification) is often incomplete, and false positives/negatives can be costly. The key bottleneck, therefore, is not only algorithmic accuracy but also industrialization: validated use cases in serial manufacturing, standardized data/metadata, rigorous uncertainty quantification, and clear links between AI outputs and decision-making (process adjustment, segregation, rework, or release).

Despite these advances, the practical value of AI-based monitoring still depends on the strength of the link between in situ signals and ex situ acceptance criteria. A model that classifies thermal anomalies with high apparent accuracy may still have limited industrial value if it has not been correlated with CT-based porosity, metallography, dimensional inspection, or mechanical properties under changing build conditions. Therefore, the critical issue is not only detection performance, but also traceability, transferability, and decision relevance. In this sense, the most promising monitoring systems are not necessarily those with the highest isolated classification scores, but those that provide stable performance across machines and materials and can be embedded into qualification or corrective-action workflows.

To complement the qualitative discussion presented above, Table 5 summarizes representative quantitative benchmarks of AI applications in metal additive manufacturing, including in situ defect detection, in situ to ex situ linkage, mechanical-property prediction, and surrogate-enabled digital twin modeling. The selected studies illustrate the diversity of sensing modalities, target variables, and performance metrics currently reported in the literature.

### 4.4. Digital Twins and Smart Manufacturing

**Figure 5:**
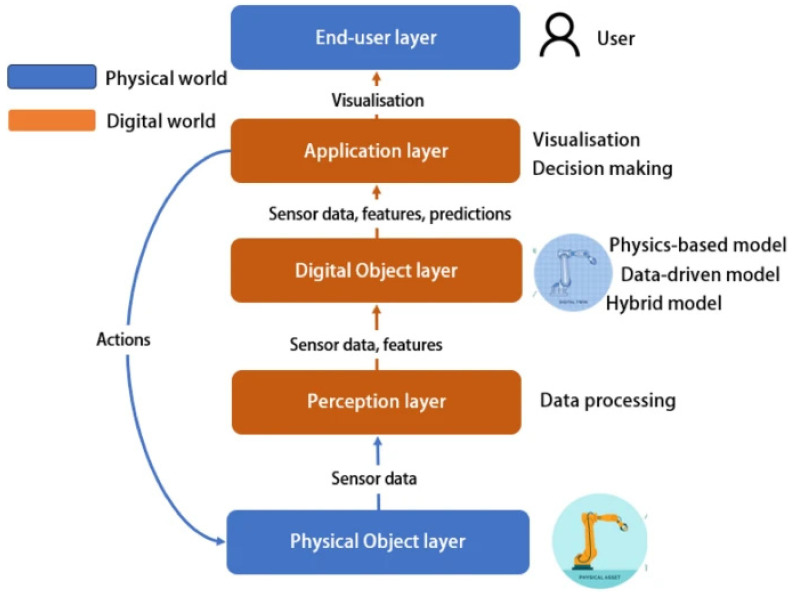
Layered framework of a digital twin, including perception, digital object, application, and end-user layers. (Reproduced from [108], licensed under CC BY-NC-ND 4.0.).

The integration of AI in MAM culminates in the concept of the digital twin: a virtual model that accurately replicates the state and behavior of the real part or process [107]. A digital twin can be understood as a digital representation of the physical process or component that is continuously updated using process data, models, and feedback information [108]. As shown in Figure 5, this concept can be interpreted as a layered architecture linking the physical object to perception, digital modeling, application, and end-user decision levels. By combining physics-based simulation with machine-learning models, the digital twin is updated in real time using sensor data. This enables fully autonomous smart manufacturing, where the machine can self-optimize its process and predict maintenance needs. The digital twin not only predicts hardness or strength before printing, but also enables predictive maintenance and part certification without extensive physical testing, closing the product life cycle in an efficient and autonomous way [109].

Table 6 provides an integrated taxonomy of how AI is being deployed across the full metal additive manufacturing (MAM) workflow, linking each application domain to representative AI methods and practical use cases. At the front end, AI supports design and optimization through generative design and topology optimization to produce lightweight, manufacturable geometries for aerospace and biomedical components. In simulation and modeling, data-driven predictors and deep-learning models act as surrogates to anticipate deformation, residual stresses, and microstructure evolution, enabling parameter refinement before fabrication. During production, quality control and monitoring rely on computer vision and multi-sensor data fusion to detect defects such as porosity, spatter, or cracking and to trigger corrective actions [110,111]. AI also underpins parameter optimization, where genetic algorithms and reinforcement learning can explore multi-objective trade-offs and enable adaptive control aimed at stabilizing melt-pool conditions. Post-build, property analysis models estimate mechanical performance and service life, supporting qualification and maintenance planning. Finally, systems integration consolidates these capabilities into digital twins and smart-manufacturing architectures, where hybrid simulation–ML models and autonomous systems connect design intent, in situ data, and production decisions to improve robustness and productivity [112].

A clearer distinction must be made between currently demonstrated digital-twin implementations and broader future visions. On the one hand, narrowly scoped digital twins have already shown practical value in specific MAM tasks. For instance, Bevans et al. [113] developed a physics- and data-integrated digital twin for LPBF of Inconel 718 that combined in situ thermal and optical tomography, experimentally validated thermal simulation, and machine learning to predict porosity, melt-pool depth, grain size, and microhardness, reporting $R^{2}$ values above 90%. This type of implementation represents a realistic, bounded use of digital twins for in situ quality assessment rather than a fully autonomous production-wide twin. On the other hand, broader reviews of digital twins in DED-Arc/W and AM consistently indicate that many published architectures remain partial, generic, or insufficiently detailed in terms of real-time integration, material modeling, and engineering implementation [114]. Therefore, the current state of the art is better described as modular and application-specific, whereas fully integrated, certifiable, and plant-scale digital twins remain a longer-term objective [115].

The current state of digital twins in metal additive manufacturing is shaped by a tension between two levels of ambition. On one side, there are narrowly scoped, well-defined twins (e.g., thermal or melt-pool twins, distortion/residual-stress twins, or in situ quality twins built on sensor signals) that operate as effective “modules” for specific tasks such as prediction, anomaly detection, or parameter adjustment. On the other side, the broader vision—an end-to-end production-chain twin that connects design, simulation, fabrication, post-processing, inspection, and in-service performance—remains largely aspirational. Real industrial integration in serial manufacturing is still limited because it requires robust data interoperability (formats, traceability, metadata), tight integration with shop-floor systems, multi-sensor and multi-machine time synchronization, and near-real-time computation without compromising process stability. In addition, validation and governance challenges persist uncertainty quantification, generalization across machines/powder lots/geometries, twin maintenance under process drift, cybersecurity, and limited availability of “ground truth” labels (CT, mechanical testing) needed to close the certification loop. As a result, the prevailing landscape today is one of partial but valuable digital twins that deliver measurable benefits in specific process windows, while fully integrated, certification-grade twins for production-scale decision-making remain relatively scarce.

**Table 6:** Application categories, key sub-applications, AI methods, and examples in MAM.

Application Category	Key Sub-Applications	AI Methods Used	Examples of Use in MAM
Design and Optimization	Generative Design	Evolutionary algorithms, generative neural networks	Creation of organic and lightweight structures for aerospace components, reducing the weight of an engine bracket [116,117].
	Topology Optimization	ML-based optimization algorithms	Material reduction in a medical implant to improve integration and reduce weight while maintaining strength [45,118].
Simulation and Modeling	Predictive process models	Neural networks, regression models (SVM, decision trees)	Prediction of deformation and residual stresses in a part before printing to adjust parameters and avoid failures [40].
	Microstructure modeling	Convolutional neural networks (CNN)	Prediction of grain structure and hardness based on scanning strategy and laser power [119,120].
Quality Control and Monitoring	Real-time defect detection	Computer vision, deep learning	Use of thermal cameras to detect porosity, spatter, or cracks in the powder bed during manufacturing [121].
	Multi-sensor monitoring	Data fusion, reinforcement learning	Combining acoustic and optical sensor data to identify anomalies and autonomously adjust parameters [122].
Parameter Optimization	Parameter planning	Genetic algorithms, neural networks	Multi-objective optimization to find the ideal scanning speed and laser power that maximize density and minimize roughness [123].
	Adaptive control	Reinforcement learning	The system learns to modulate laser power and speed in real time to maintain an optimal, constant melt temperature [124].
Property Analysis	Mechanical property prediction	Neural networks, regression algorithms	Prediction of hardness, tensile strength, and fatigue strength based on manufacturing data [125].
	Service life prediction	Advanced regression models	Estimation of part lifetime (e.g., a turbine or implant) for predictive maintenance applications [126].
Systems Integration	Digital twins	Hybrid models (simulation + ML)	Creation of a virtual replica of a part that predicts performance under different loads and service temperatures [127].
	Smart manufacturing	AI-based autonomous systems	A fully interconnected factory where 3D printers adjust processes and communicate with each other to optimize production [128].

## 5. Critical Comparison and Emerging Trends

AI applications in metal additive manufacturing show uneven maturity. The most established approaches are supervised learning models used for process–property prediction and computer-vision pipelines for layerwise monitoring, particularly in PBF-LB/M, where instrumentation is more standardized and data are more abundant. Surrogate models trained on simulation outputs are also comparatively mature for offline screening of parameter windows and distortion trends, because they fit well within existing qualification workflows. In contrast, reinforcement learning for closed-loop control, physics-informed learning for robust extrapolation, and manufacturing feasibility-constrained generative design remain predominantly research topics, mainly due to the difficulty of safe deployment, the need for high-fidelity and well-labeled datasets, and the complexity of integrating physics, sensing, and control within industrial constraints.

Current limitations are dominated by data and standardization gaps. Datasets are typically small, fragmented, and proprietary, with heterogeneous formats and incomplete metadata (machine state, calibration, material pedigree, environment, maintenance history). Ground truth for defects is expensive and often indirect, and model performance frequently degrades under dataset shift when changing machine, material batch, shielding conditions, or geometry. These issues translate into limited generalization, insufficient uncertainty quantification for decision-making, and difficulty mapping sensor signatures to acceptance criteria required for certification. In addition, interoperability barriers—closed machine interfaces, non-uniform sensor stacks, and inconsistent reporting metrics—slow down replication and technology transfer. A distinction should be made between process capability in general and suitability for a given application class. MAM already demonstrates robust manufacturing performance in selected non-critical or moderately critical applications, especially where geometry complexity, customization, or repair justify its use and where qualification can be performed within bounded process windows. However, in highly critical sectors such as aerospace propulsion, pressure-containing energy components, or safety-relevant medical implants, the required level of repeatability, traceability, and evidence remains substantially higher. Under these conditions, the value of AI is not merely to improve prediction accuracy, but to help bridge the gap between technically feasible manufacturing and certifiable manufacturing.

For qualification-oriented use, uncertainty quantification should move from a general recommendation to a practical design requirement. In AI-based MAM workflows, calibrated uncertainty may be estimated through Bayesian deep learning, deep ensembles, or conformal prediction frameworks, depending on the sensing modality and decision latency constraints. The key issue is not uncertainty reporting alone, but its propagation into action thresholds: high-confidence predictions may support release or parameter continuation, intermediate-confidence outputs may trigger additional inspection or rework, and low-confidence or out-of-distribution predictions should lead to conservative intervention, such as parameter correction, part segregation, or rejection. In this sense, uncertainty-aware AI is essential not only for scientific robustness, but also for operational decision-making under certification constraints.

Integration with IoT and edge computing is a clear near-term trend. Multi-sensor fusion architectures combining optical, thermal, acoustic, and electrical signals can improve robustness, while edge inference reduces latency and bandwidth requirements for in-process alarms and corrective actions. A practical architecture is hierarchical: edge devices handle real-time detection and coarse control, whereas cloud/HPC resources support fleet learning, periodic retraining, and governance. The medium-term convergence point is the digital twin, where hybrid physics–ML models are continuously updated with sensor streams to estimate latent states (thermal history, melt pool stability, residual stress proxies) and support risk-aware decisions. For this integration to become routine, the field will need shared data schemas, calibrated uncertainty reporting, and secure pipelines that respect industrial IP and cybersecurity constraints. In practical terms, such digital-twin frameworks may integrate in situ monitoring signals (e.g., melt pool imaging, acoustic emissions, or thermal signatures) with ex situ validation data obtained from computed tomography, density measurements, or mechanical testing. These ex situ measurements provide ground-truth references that allow periodic model recalibration and traceability within qualification workflows, enabling AI models to evolve alongside the manufacturing process while maintaining certification-relevant evidence.

While the concept of autonomous additive manufacturing systems is frequently discussed in the literature, current industrial implementations remain more limited in scope. Most operational digital twin frameworks focus on monitoring, prediction, and decision support rather than fully autonomous control of the manufacturing process. For example, recent implementations integrate in situ sensing with predictive models to estimate part quality and support qualification workflows in laser powder bed fusion environments, while maintaining human supervision and external validation steps [111,113,115]. These developments suggest that the near-term role of AI and digital twins in MAM is likely to augment operator decision-making and process understanding rather than to enable fully autonomous manufacturing systems.

For certification-oriented use, AI models should be understood as part of a broader validation chain rather than as stand-alone acceptance tools. A critical requirement for industrial adoption is that AI models be validated not only against experimental observations, but against qualification-relevant manufacturing outcomes. In this context, the link between in situ sensing and certified part acceptance must be explicitly established through ex situ evidence such as computed tomography (CT) inspection, density measurements, metallography, dimensional verification, hardness testing, tensile performance, and, where relevant, fatigue testing. This is especially important in highly regulated sectors, where predictive accuracy alone is insufficient unless it is accompanied by repeatability, uncertainty quantification, traceable metadata, and version-controlled model-governance procedures compatible with standards-based qualification. Accordingly, AI should be interpreted as an enabling layer within a broader validation architecture, supporting inspection prioritization, process correction, and decision-making, but not replacing the certification evidence required for part release.

Current literature also shows a clear concentration of AI applications in fusion-based metal additive manufacturing processes, particularly laser powder bed fusion and directed energy deposition, largely due to their widespread industrial adoption and the availability of high-resolution in situ monitoring data [5,19,34]. Despite the strong potential of AI in metal additive manufacturing, its integration also involves important risks that should not be overlooked. Many reported models are trained on limited datasets generated under narrow process windows, increasing the risk of overfitting, poor generalization, and unreliable predictions when process conditions, materials, machines, or geometries change. In addition, sparse data coverage may lead to misplaced confidence in model outputs, especially when rare defects or edge-case conditions are underrepresented in the training set. Another important limitation is that purely data-driven models may fail to capture the strongly coupled multi-physics nature of metal additive manufacturing, where thermal gradients, melt-pool dynamics, phase transformations, residual stress development, and microstructural evolution interact across multiple length and time scales. Under these conditions, apparently accurate predictions may still be physically incomplete or insufficiently robust for industrial deployment. These risks highlight the importance of hybrid physics–ML approaches, uncertainty quantification, domain-aware validation, and strict control of the operational envelope within which AI models are applied.

Across the reviewed studies, a clear pattern emerges: although artificial intelligence has shown strong potential in metal additive manufacturing, many reported advances remain highly dependent on narrowly defined datasets, specific machine configurations, and controlled laboratory conditions. This limits direct transferability across materials, geometries, and process platforms. In particular, a recurring weakness in the literature is the limited use of robust external validation, as many models are assessed on data generated under restricted experimental conditions without sufficient evidence of generalization under production variability. Similarly, although high predictive performance is frequently reported, the practical value of such models is often constrained by insufficient interpretability, weak linkage to process physics, and limited integration with qualification and certification requirements.

Another important trend is that the most promising studies are not necessarily those relying on increasingly complex algorithms, but rather those combining data-driven methods with process knowledge, multimodal sensing, and physically meaningful validation strategies. In this sense, hybrid physics–ML frameworks appear particularly relevant for the future of MAM, as they offer a more credible route toward robustness, extrapolation capability, and industrial trustworthiness. Therefore, the current challenge is no longer only to improve prediction accuracy, but to develop AI systems that are transferable, explainable, and verifiable under realistic manufacturing conditions.

## 6. Conclusions

This review set out to examine how artificial intelligence is being applied across the principal stages of metal additive manufacturing, with particular attention to process understanding, monitoring, prediction, and quality-oriented decision support. Taken together, the reviewed literature shows that the field is progressing from isolated proof-of-concept studies toward more integrated and deployment-aware frameworks. However, the main challenge is no longer to demonstrate that AI can improve individual tasks, but to ensure that these methods are transferable, explainable, and compatible with validation and qualification requirements under realistic manufacturing conditions.

From this perspective, the main value of AI in MAM lies not only in improving prediction accuracy, but in enabling more efficient qualification, more consistent process operation, and more informed quality assurance. At the same time, the review also shows that many currently reported approaches remain constrained by narrow datasets, limited external validation, and insufficient attention to certification-oriented deployment. Therefore, the real progress of the field should be measured less by isolated performance gains and more by the development of robust, traceable, and industrially credible AI frameworks.

AI contributes to MAM not only by improving prediction and monitoring capabilities, but also by reshaping how qualification, process consistency, and quality assurance can be approached in practice. Across the reviewed literature, the most valuable contributions are not simply those reporting high predictive accuracy, but those enabling more efficient parameter screening, earlier defect awareness, faster thermal–mechanical estimation, and design decisions that better integrate manufacturability constraints. In this sense, AI is progressively moving from isolated support functions toward a broader enabling role in closed-loop control and digital-twin-based production. Nevertheless, the review also shows that this transition remains uneven, as many reported solutions are still validated under narrow conditions and have not yet demonstrated robust industrial portability. From a deployment perspective, dataset shift should be treated as an expected operating condition rather than as an exception. Relevant sources of shift include powder-lot variability, optics contamination, shielding-gas changes, machine-to-machine differences, recoater wear, and geometry-dependent thermal histories. As a result, model portability should not be assumed unless revalidation has been performed under the target machine, material, and process configuration.

Future challenges are mainly methodological and infrastructural. Interoperability remains a prerequisite and requires standard datasets, shared metadata conventions, sensor synchronization practices, and machine–software interfaces that make solutions portable. Validation and certification demand uncertainty-aware models, traceable ground truth, drift monitoring, and audit-ready model governance. Sustainability should also be addressed explicitly by incorporating energy use, material efficiency, rework rates, and life-cycle indicators into optimization objectives rather than focusing only on geometric or mechanical metrics. Finally, secure data sharing and privacy-preserving learning will be necessary to scale AI across organizations without compromising intellectual property.

For researchers, the main priorities include reproducible benchmarking, including cross-machine and cross-material validation, rigorous leakage control, standardized reporting, and uncertainty quantification aligned with acceptance criteria. Methodologically, hybrid physics–ML approaches and domain adaptation appear more promising than further gains based only on single-dataset accuracy. For industry, the most realistic path is incremental deployment, beginning with high-return applications such as monitoring and parameter screening, supported by robust data governance, calibration routines, clearly defined decision thresholds, and formal model lifecycle management. This approach can deliver near-term value while establishing the basis for future digital-twin-driven certification and adaptive control.

## Figures and Tables

**Table 1:** Comparison of main Metal Additive Manufacturing technologies.

Technology	Feedstock	Main Advantages	Limitations	Representative Applications (Non-Exclusive)
PBF-LB/M	Metal powder	High accuracy, good surface finish, high density	Limited volume, long build times, high costs	Aerospace, biomedical implants, high-precision components [29,30]
PBF-EB/M	Metal powder	High melting speed, suitability for reactive materials (Ti, Al)	Requires high vacuum, lower resolution than PBF-LB/M	Aerospace, turbines, titanium components [31,32]
DED-LB/M or DED-EB/M	Metal powder	Part repair, localized deposition, medium-size parts	Lower accuracy than PBF, machining often required	Aerospace repair, molds, functional prototypes [33,34]
DED-ARC/W	Metal wire	High deposition rate, lower costs, large-part fabrication	Low resolution, intensive post-processing required	Large structures, shipbuilding, energy, defense [20,35]
Binder Jetting/M	Metal powder	High productivity, complex parts without supports	Lower mechanical properties, infiltration or sintering required	Medium series, automotive, tooling [36,37]

**Table 3:** AI applications in AM: representative algorithms and reported results.

Application	Typical Algorithms	Representative Studies and Outcomes
Topology and optimization	Deep generative models, CNN, GAN, physics-informed networks	Topology generation with GANs and dual discriminators to satisfy mechanical and geometric constraints; mechanically valid 2D structures [57]
Generative design	GANs, advanced 2D/3D models, conditional generation	Design frameworks incorporating casting/molding constraints to improve manufacturing feasibility and reduce redesign cycles [58]
Parameter optimization	Regression, ensembles, ANN, hybrids (surrogate + GA/metaheuristics)	Polynomial regression predicted compressive strength with R^2^ = 0.88 and 3.44% error, outperforming Taguchi in an FFF case [61]
Quality prediction and monitoring	CNN for images, RF/XGBoost, tuned ANN	In situ anomaly detection in metals and polymers; improved surface and strength prediction using supervised ensembles [62,64]

**Table 5:** Representative quantitative benchmarks of AI applications in metal additive manufacturing.

Ref.	Process	Input Mode	Task	Model	Metric(s)	Latency/Speedup
Pak et al. [89]	PBF-LB/M	In situ thermal imaging	Ex situ porosity quantification and localization	CNN for quantification; Video Vision Transformer for localization	(R^2^ = 0.57) for porosity quantification; average IoU = 0.32 for porosity localization	Not explicitly reported for model inference. Pyrometry acquired at 6–7 kHz, with 1000 frames per layer before filtering
Luo et al. [105]	PBF-LB/M	In situ photodiode signals converted to image-like representations	Mechanical-property prediction (UTS and elongation to fracture)	Transfer-learning/DCNN-based regression	98.7% average cross-validation accuracy, (R^2^ = 0.89) for UTS; 93.1% average cross-validation accuracy, (R^2^ = 0.96) for elongation to fracture	Hardware accelerated inference speeds mentioned, but no exact latency value
Chen et al. [78]	DED-LB/M	Acoustic + coaxial visible-spectrum vision	Real-time, location-dependent defect detection	Hybrid CNN for multimodal fusion	98.5% defect-prediction accuracy	Real-time defect detection; 10 Hz was mentioned in your target formulation, but I could not verify that frequency from the accessible snippet
Liu et al. [106]	PBF-LB/M	Physics-generated melt-pool temperature fields with DT assimilation framework	Digital-twin surrogate modeling and process-window generation	Fourier Neural Operator (FNO)	Relative (L_2) test error: 0.82% (in-plane section) and 0.96% (scan-direction section)	1000 parameter combinations evaluated in about 20 s on a single GPU; equivalent full physical simulations reported as 14 days on the same machine with 48 CPU cores

## Data Availability

No new data were created or analyzed in this study. Data sharing is not applicable to this article.
